# Epidemiology and outcomes of anaphylaxis-associated out-of-hospital cardiac arrest

**DOI:** 10.1371/journal.pone.0194921

**Published:** 2018-03-26

**Authors:** Sun Young Lee, Seung Chul Lee, Sang Do Shin, Kyoung Jun Song, Young Sun Ro, Jeong Ho Park, So Yeon Kong

**Affiliations:** 1 Department of Emergency Medicine, Seoul National University College of Medicine, Seoul, Republic of Korea; 2 Laboratory of Emergency Medical Services, Seoul National University Hospital Biomedical Research Institute, Seoul, Republic of Korea; 3 Department of Emergency Medicine, Dongguk University Ilsan Hospital, Goyang-si, Republic of Korea; Azienda Ospedaliero Universitaria Careggi, ITALY

## Abstract

**Background:**

Understanding the epidemiological characteristics of anaphylaxis-associated out-of-hospital cardiac arrest (OHCA) is the first step toward developing preventative strategies and optimizing care systems. We aimed to describe and compare epidemiological features and clinical outcomes among patients with anaphylaxis-associated OHCAs according to causative agent groups.

**Methods:**

We identified emergency medical service (EMS)-treated anaphylaxis-associated OHCA patients from a nationwide OHCA registry between 2008 and 2015. We compared epidemiological characteristics and outcomes according to causal agents (a natural agents group and an iatrogenic agents group) and evaluated temporal variability in incidence. Multivariate logistic regression analysis was performed to compare survival to discharge between causative agent groups.

**Results:**

During the study period (8 years), the total number of anaphylaxis-associated OHCAs was 233. A total of 224 eligible cases were included in the analysis. There were 192 patients (85.6%) in the natural agents group and 32 patients (14.3%) in the iatrogenic agents group. There was significant diurnal and seasonal variability in the frequency of anaphylaxis-associated OHCAs (*p* values<0.01 for both), with the highest incidences occurring during the day (7:01 am to 3 pm; 64.6%) and in summer (June to August, 48.7%). Compared with the natural agents group, the adjusted odds ratio (AOR) for survival to discharge in the iatrogenic agents group was statistically insignificant (AOR 3.61, 95% CI 0.86 to 15.06).

**Conclusion:**

The incidence of anaphylaxis-associated OHCA is considerably low, and significant temporal variability, with a peak during the day and in summer, is evident. Anaphylaxis-associated OHCA is more common by natural agents than by iatrogenic agents, but no difference in the survival-to-discharge rate is evident.

## Introduction

Anaphylaxis is a potentially life-threatening allergic reaction. Because anaphylaxis can rapidly become fatal, avoiding the allergen and obtaining early treatment are critical for preventing fatal outcomes [1–3]. Guidelines universally recommend that patients who have had an anaphylactic reaction should avoid an allergen and carry an epinephrine auto-injector (known as an epi-pen) [3, 4]. However, the auto-injector is not widely used, especially when anaphylaxis occurs outside of the hospital [5]. In general, anaphylaxis does not occur if the triggering allergen does not work, so identifying and avoiding the dangerous allergen is important, especially for allergic reactions occurring outside of the hospital, where the epi-pen is rarely used. However, despite the increasing number of allergic patients in modern society [6, 7], neither the most common dangerous allergens nor the characteristics of fatal anaphylaxis are well known.

In previous studies, the known life-time prevalence of anaphylaxis has been estimated to be approximately 0.05 to 2.00%, and this number is growing [8–10]. In developed countries, such as the United States (U.S.) and United Kingdom (U.K.), the incidence of anaphylaxis is 40 to 500 per million person-years [6, 8, 9], and according to Spanish studies, more than 5,000 patients have been hospitalized for anaphylaxis over a period of 15 years [10]. Regarding fatal cases, anaphylaxis-associated mortality is estimated at 0.63 to 0.76 per million people, and other studies have reported that the mortality rate for patients with severe anaphylaxis reactions is from 0.65 to 2.0% [6, 8, 11, 12]. However, these results were based on a small region, a specific population group or hospitalized patients only. No studies have investigated the incidence of fatal anaphylaxis in a community with a large population. Furthermore, despite the need for knowledge regarding the characteristics of allergens to prevent fatal anaphylaxis, the allergens that are most dangerous and the usual timing of fatal anaphylaxis in the community are unclear. Accurate epidemiological and outcome data on anaphylaxis-associated out-of-hospital cardiac arrest (OHCA) are needed to establish a preventative strategy and community education program.

The aim of this study was to investigate the epidemiology and clinical outcomes of anaphylaxis-associated OHCA in the community using a nationwide OHCA registry database.

## Materials and methods

This is a population-based observational study based on a nationwide OHCA registry database in Korea.

### Study setting

Korea has a population of approximately 50 million people over a 99,000-km^2^ area of land. The Korea Emergency Medical Service (EMS) is a single-tiered, government-operated system. Ambulance services with an automated external defibrillators (AEDs) are operated by 16 provincial fire departments (FDs). All emergency medical technicians (EMTs) can provide basic life support (BLS) to treat cardiac arrest patients at the scene but cannot give medication, such as epinephrine, for advanced cardiac life support (ACLS). EMTs transport all OHCA patients to an emergency department (ED) according to an EMS cardiopulmonary resuscitation (CPR) protocol because only doctors can declare death in Korea.

### Study data sources

The nationwide OHCA registry contains data for all cardiac arrest patients transported by EMS in Korea. The registry was developed by the Korea Centers for Disease Control and Prevention (CDC) in 2006. The EMS run sheet, EMS cardiac arrest registry for the Utstein factor, and dispatcher CPR registry were merged with medical record review data, including hospital care and survival outcomes. This merged data source was constructed according to an international OHCA database guideline [13], and stepwise data quality management was conducted. First, a trained EMT records the EMS data according to the data dictionary of the EMS record variable, and the medical director of each FD performs quality management. Second, the Korea CDC trains medical record reviewers and conducts medical record surveys. Afterward, the data quality management committee reviews the collected data and provides feedback every month. Detailed information and the collection process of the nationwide OHCA registry has been reported previously [14, 15].

In this OHCA registry, the cause of arrest was classified into two categories: disease and non-disease. The disease group was subclassified as cardiogenic; respiratory; non-traumatic bleeding; terminal disease, including cancer and sudden infant death syndrome (SIDS); and other diseases. The non-disease group was subclassified as traffic accident, fall, blunt trauma, penetrating injury, gunshot, burn, asphyxia, drowning, hanging, poisoning, others, and unknown. Anaphylaxis is included in the “others” classification of the non-disease group and is recorded in the text as “anaphylaxis”.

### Study subjects

All patients with OHCAs who were transported by the ambulance service between January 1, 2008 and December 31, 2015 were selected. Anaphylaxis-associated OHCA patients were identified when the presumed cause of arrest was recorded as anaphylaxis in the OHCA database. Patients who did not receive resuscitation or were unable to determine the causative agents were excluded.

### Main outcomes

The primary outcome was survival to discharge. The secondary outcome was a good neurological recovery at discharge from a hospital, which was defined as a Cerebral Performance Category score of 1 (good cerebral performance; able to work) or 2 (moderate cerebral disability; able to perform daily activities independently).

### Variables and measurements

We collected variables according to the Utstein template. These variables included age, gender, urbanization level (metropolitan city > 1 million population), the location of the event (public, private, ambulance, and unknown), event location (inside, outside, and unknown), past medical history (diabetes mellitus, hypertension, heart disease, and stroke), whether the event was witnessed, the primary electrocardiogram (ECG) rhythm on scene (shockable, including VF and pulseless VT; non-shockable, including PEA and asystole), bystander CPR (yes or no), the EMS response time interval (interval from call to EMS arrival at the scene), the level of ED (level I, II, and III), the achievement of a prehospital return of spontaneous circulation (ROSC), survival to discharge, and good neurological recovery, namely, Cerebral Performance Category 1 or 2.

The time variables (time of arrest and season of arrest) were defined as the time the emergency call was received. All cases were classified according to the categories used in the previous study [16]: (1) daytime (7:01 am to 3 pm), evening (3:01 pm to 11 pm), and overnight (11:01 pm to 7:00 am); and (2) winter (December to February), spring (March to May), summer (June to August), and autumn (September to November).

The main factor of interest was the causative agent of anaphylaxis-associated OHCA. There are three types of causative agents in the worldwide anaphylaxis guidelines: an insect bite (bite-induced anaphylaxis), food, and iatrogenic agents (drugs) [3, 4, 17]. Because the number of food agents is small in this study, based on the classification used by the World Allergy Organization (WAO), we classified the causative agents into two categories: natural agents (insect stings and food) and iatrogenic agents (drugs and injections) [4]. We identified the causative agents through the text review of the OHCA registry database. If the precise causative agent was recorded in the database, we recorded the agent. If not, we estimated the presumed substance through the circumstances recorded in the database.

### Statistical analysis

A descriptive analysis was conducted to describe the distribution of variables. Categorical variables were described as the number and percentage (%), and continuous variables were described as the medians and quartiles. Chi-square tests or Wilcoxon rank sum tests were used to compare each variable.

The hourly and seasonal frequency of the anaphylaxis-associated OHCA incidence are shown graphically. Temporal variability was compared with the Chi-square test.

An adjusted odds ratio (AOR) and 95% confidence interval (95% CI) for each study outcome were calculated with a multivariable logistic regression analysis. Adjustments were performed for potential confounders, including age, gender, metropolis, location, whether the event was witnessed, past medical history (diabetes mellitus, hypertension, and heart disease), time of day of the arrest, and the season of arrest. Stroke in the previous medical history was not included as an adjusted variable because this variable was zero in the iatrogenic agents group.

A two-tailed p<0.05 was considered statistically significant. All statistical analyses were conducted using SAS software, version 9.4 (SAS Institute Inc., Cary, NC, USA).

### Ethics statement

This study was approved by the institutional review boards of the Seoul National University Hospital (IRB No. H1103-153-357). Informed consent by the individual patients was waived.

## Results

### Demographic findings

**Fig 1:**
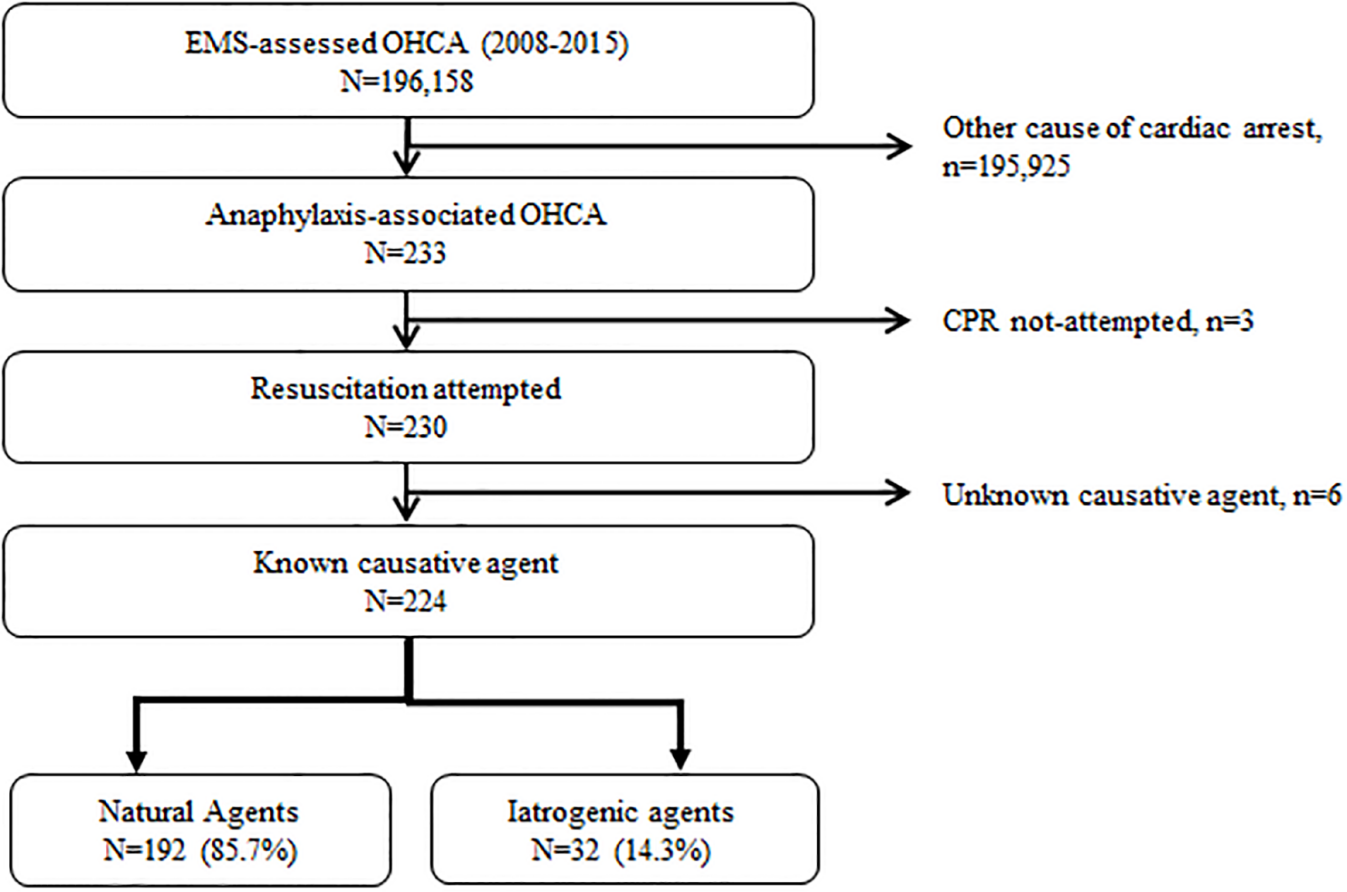
Patient enrollment flowchart.

Of the 196,158 OHCAs treated by EMS during the study period (2008 to 2015, 8 years), the total number of anaphylaxis-associated OHCAs was 233 (0.12%). A total of 224 eligible anaphylaxis-associated arrest patients were analyzed, after excluding patients without resuscitation attempts (n = 3) and with unknown causative agents (n = 6). Of the 224 patients, 192 (85.7%) were allocated to the natural agents group, and 32 (14.3%) were allocated to the iatrogenic agents group (Fig 1).

The demographics of the study groups are presented in Table 1. The natural agents group comprised more males, younger patients, non-metropolitan cases, occurrences in public locations, and outdoor incidents than the iatrogenic agents group (all *p* value<0.01). The number of cases with witnesses, shockable rhythms and shorter response time intervals was higher in the iatrogenic agents group (*p* value<0.01, 0.04, and <0.01, respectively) than in the natural agents group. Prehospital ROSC, survival to discharge and good neurological recovery were more frequent in the iatrogenic agents group (9 (28.1%), 12 (37.5%), and 8 (25.0%)) than in the natural agents group (14 (7.3%), 22 (11.5%), and 8 (4.2%)) (all *p* value<0.01) (Table 1).

**Table 1:** Demographics of anaphylaxis-associated OHCA according to the causative agent group.

	Total		Natural		Iatrogenic		*p value*
	N	%	N	%	N	%	
**All**	224	100.0	192	100.0	32	100.0	
**Gender**							<0.01
** Female**	62	27.7	37	19.3	25	78.1	
** Male**	162	72.3	155	80.7	7	21.9	
**Age, years**							<0.01
** 19–39**	15	6.7	9	4.7	6	18.8	
** 40–49**	43	19.2	40	20.8	3	9.4	
** 50–59**	60	26.8	55	28.6	5	15.6	
** 60–69**	67	29.9	56	29.2	11	34.4	
** 70–79**	34	15.2	30	15.6	4	12.5	
** 80-**	5	2.2	2	1.0	3	9.4	
**Median(IQR)**	59 (50–67)		58.5 (50–66.5)		62.5 (47–69.5)		
**Metropolis**							<0.01
** Non-metropolitan**	178	79.5	165	85.9	13	40.6	
** Metropolitan**	46	20.5	27	14.1	19	59.4	
**Location**							<0.01
** Public**	122	54.5	122	63.5	0	0.0	
** Private**	66	29.5	36	18.8	30	93.8	
** Ambulance**	13	5.8	12	6.3	1	3.1	
** Unknown**	23	10.3	22	11.5	1	3.1	
**Event location**							<0.01
** Indoors**	45	20.1	15	7.8	30	93.8	
** Outdoors**	119	53.1	118	61.5	1	3.1	
** Unknown**	60	26.8	59	30.7	1	3.1	
**Time of arrest**							0.43
** Daytime (7:01–15:00)**	142	63.4	124	64.6	18	56.3	
** Evening (15:01–23:00)**	70	31.3	57	29.7	13	40.6	
** Overnight (23:01–07:00)**	12	5.4	11	5.7	1	3.1	
**Season of the arrest**							<0.01
** Winter (December-February)**	11	4.9	3	1.6	8	25.0	
** Spring (March-May)**	26	11.6	12	6.3	14	43.8	
** Summer (June-August)**	109	48.7	106	55.2	3	9.4	
** Autumn (September-November)**	78	34.8	71	37.0	7	21.9	
**Past medical history**							
** Diabetes mellitus**	28	12.5	25	13.0	3	9.4	0.56
** Hypertension**	52	23.2	45	23.4	7	21.9	0.85
** Heart disease**	12	5.4	8	4.2	4	12.5	0.05
** Stroke**	3	1.3	3	1.6	0	0.0	0.48
**Witnessed**							<0.01
** Yes**	164	73.2	133	69.3	31	96.9	
**Primary ECG**							0.04
** Non-shockable**	204	91.1	178	92.7	26	81.3	
** Shockable**	20	8.9	14	7.3	6	18.8	
**Bystander CPR**							0.04
** Yes**	16	7.1	16	8.3	0	0.0	
**EMS response time interval, min**							<0.01
** Median (IQR)**	11 (7–17)		13 (8–19)		6 (5–8)		
**ED Level**							0.05
** Level I**	24	10.7	20	10.4	4	12.5	
** Level II**	80	35.7	63	32.8	17	53.1	
** Level III**	120	53.6	109	56.8	11	34.4	
**Outcome**							
** Prehospital ROSC**	23	10.3	14	7.3	9	28.1	<0.01
** Survival to discharge**	34	15.2	22	11.5	12	37.5	<0.01
** Good neurological recovery**	16	7.1	8	4.2	8	25.0	<0.01

Abbreviations: OHCA; out-of-hospital cardiac arrest; IQR, interquartile range; ECG, electrocardiogram; CPR, cardiopulmonary resuscitation; EMS, emergency medical service; ROSC, return of spontaneous circulation; ED, emergency department

Table 2 shows the detailed information of the causative agents. Of the 192 OHCAs in the natural agents group, 163 (84.9%) were caused by bee stings, including four bee venom acupuncture cases. Five cases of food allergens were identified (2.6% in the natural agents group). Nine of the iatrogenic agents had exact identifications (anesthetics, 3; antibiotics, 3; and NSAIDs, 3) (Table 2).

**Table 2:** Causative agents of anaphylaxis-associated OHCA.

	N	%	Text
**Natural**			
** Total**	192	100.0	
** Bee sting**	163	84.9	bee venom acupuncture (4)
** Ant bite**	1	0.5	
** Centipede bite**	3	1.6	
** Unspecified bite**	20	10.4	
** Food**	5	2.6	sashimi (1), plum (1), peach (1), steamed silkworm chrysalis (1), dog meat (1)
**Iatrogenic**			
** Total**	32	100.0	
** Anesthesia**	3	9.4	lidocaine (1)
** Antibiotics**	3	9.4	
** NSAIDs**	3	9.4	diclofenac (1), unifenac (1), dicknol (1)
** Unspecified**	23	71.9	nutritional agent (1), spinal injection (1), joint injection (1), common cold medication injection (1)

Abbreviations: OHCA; out-of-hospital cardiac arrest; NSAID, nonsteroidal anti-inflammatory drug

### Main analysis

#### Diurnal and seasonal variability in anaphylaxis-associated OHCA incidence

**Fig 2:**
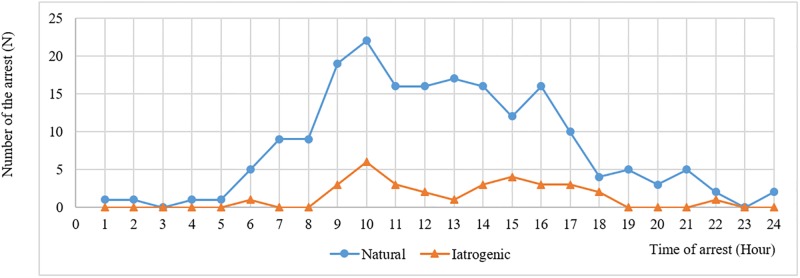
Diurnal variability of anaphylaxis-associated OHCA.

Emergency calls for anaphylaxis-associated OHCA occurred more frequently during the day (63.4%) than during the evening (31.3%) or overnight (5.4%) (Table 1). Two peaks in occurrence were observed, with a large peak from 9 to 11 am and a smaller peak from 3 to 5 pm. The peak was more prominent for natural agents. Overnight arrests were rare in both groups (Fig 2).

**Fig 3:**
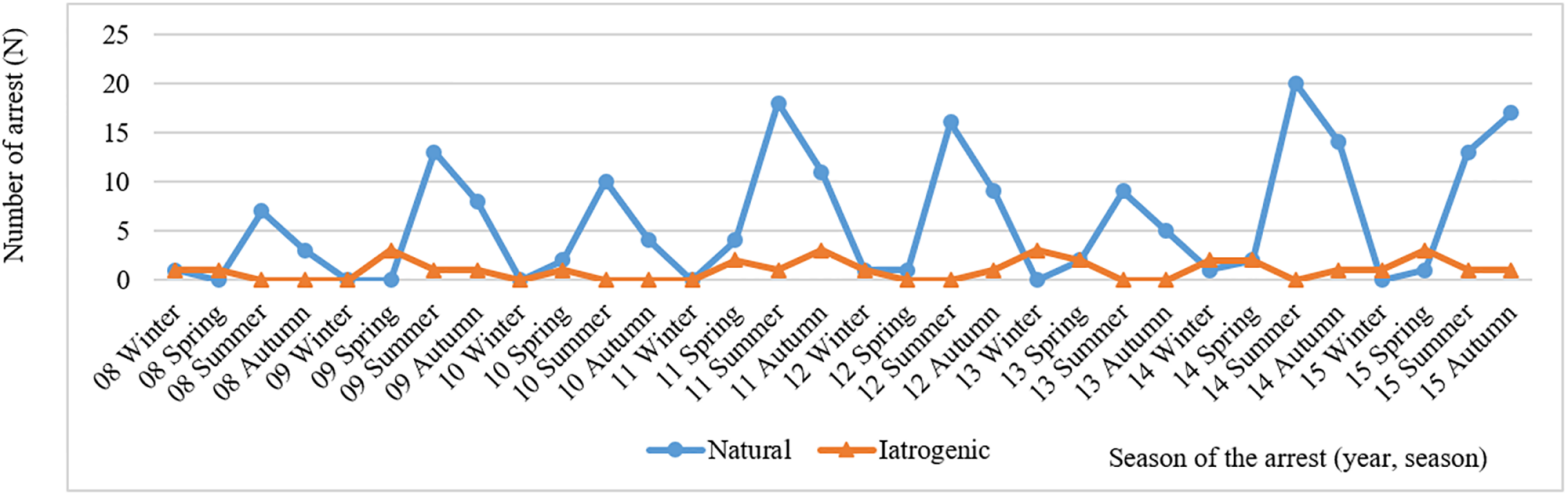
Seasonal variability of anaphylaxis-associated OHCA.

The highest incidence of anaphylaxis-associated OHCA occurred during the summer season, especially from June to August (Table 1). This seasonal difference is mainly due to the seasonal variations in natural agents. No significant seasonal variations in the iatrogenic agents were evident. Similar trends were observed every year from 2008 to 2015 (Fig 3).

#### Multivariable logistic regression analysis of study outcomes by causative agent group

Compared to the natural agents group, the AOR (95% CI) for survival to discharge was 3.97 (1.39 to 11.37) for the iatrogenic agents group in the age- and gender-adjusted model (Model I). Model II was constructed by adding the demographic variables (metropolis, location of arrest, whether the event was witnessed, and past medical history) to the Model I, and Model III was constructed by adding the time variables (time of arrest and season of arrest) to Model II. In the final model (Model III), after adjusting for all variables, the AOR for survival to discharge was statistically non-significant (AOR 3.61, 95% CI 0.86 to 15.06) (Model III).

For good neurological outcome, the AOR (95% CI) was 4.02 (1.07 to 15.15) for the iatrogenic agents group in the age- and gender-adjusted model (Model I). After adjusting for all variables, the AOR (95% CIs) was 8.27 (1.39 to 49.17) (Model III) (Table 3).

**Table 3:** Logistic regression analysis of survival to discharge and good neurological outcome by causative agent group.

	Total	Outcome		Model I*			Model II**			Model III***		
		N	%	OR	95% CI		OR	95% CI		OR	95% CI	
**Survival to discharge**												
** Total**	224	34	15.2									
** Natural**	192	22	11.5	1.00			1.00			1.00		
** Iatrogenic**	32	12	37.5	3.97	1.39	11.37	2.76	0.84	9.12	3.61	0.86	15.06
**Good neurological outcome**												
** Total**	224	16	7.1									
** Natural**	192	8	4.2	1.00			1.00			1.00		
** Iatrogenic**	32	8	25.0	4.02	1.07	15.15	4.19	0.95	18.54	8.27	1.39	49.17

Abbreviations: OR, odds ratio; CI, confidence interval

*Adjusted for age, gender

**Adjusted for age, gender, metropolis, location, and past medical history (diabetes mellitus, hypertension, and heart disease)

***Adjusted for age, gender, metropolis, location, whether the event was witnessed, past medical history (diabetes mellitus, hypertension, and heart disease), time of arrest and season of arrest

## Discussion

To our knowledge, this is the first nationwide observational study of anaphylaxis-associated OHCA. In Korea, anaphylaxis-associated OHCA occurred in 233 cases over 8 years. Anaphylaxis-associated OHCA occurred most frequently in during the day and during summer. This is mainly due to seasonal variations in arrest caused by natural agents. No significant differences were evident in terms of survival to discharge between the natural and iatrogenic agent groups.

Because anaphylaxis occurs suddenly, investigations of the incidence of anaphylaxis in the community are difficult. According to previous studies, the incidence of anaphylaxis is less than 1%, and mortality is low [18]. In a meta-analysis conducted in the U.S., the incidence of anaphylaxis in the general population was reported to be 1.2 to 16.8% of the total U.S. population [19]. According to Korean studies using the National Health Insurance (NHI) claims data, the incidence of anaphylaxis continuously increases every year regardless of the sex or age group [7]. Anaphylaxis-related mortality was less than 1 per million in studies conducted in the U.S. and U.K. [6, 19–21]. Although a comparison of the results of this study to those of previous studies, which were derived from diverse population groups, is difficult, 233 anaphylaxis-associated cases in this study were found among the 196,158 EMS-assessed OHCAs that occurred over 8 years. These cases account for 0.12% of all OHCAs in Korea. This study is the first to investigate the proportion of OHCAs that comprises anaphylaxis, and the analysis has advantages in that it uses a nationwide OHCA database.

The epidemiological characteristics of anaphylaxis-associated OHCA in this study are described below. Anaphylaxis-associated OHCAs due to natural agents more frequently occurred in non-metropolitan areas and had longer EMS response times than anaphylaxis-associated OHCAs due to iatrogenic agents. However, there was no significant difference in the survival outcome when the confounders were adjusted. This may be due to the overall good survival rate of anaphylaxis-associated OHCA regardless of the causative agent [22]. Twelve cases in the study population had heart disease, and the type of heart disease was identified in 5 patients; 3 were in the natural agents group, and 2 were in the iatrogenic agents group. Heart disease in the natural agents group comprised angina, congestive heart failure, and a known heart procedure (coronary stent or CABG). Heart disease in the iatrogenic agents group comprised angina and arrhythmia. Acute coronary syndrome (ACS) can occur due to a strong immune reaction during anaphylaxis, and this condition is known as Kounis syndrome [23]. An anaphylaxis-associated OHCA may be associated with Kounis syndrome. However, in the database used in this study, we could not investigate whether the patients complained of chest pain before the arrest or whether they had coronary lesions. The proportion of cases with Kounis syndrome in the study population is unknown.

We investigated the exact causative agents of fatal anaphylaxis in the community. According to previous studies, the most common cause of anaphylaxis is food. More than 50% of anaphylaxis incidents are caused by food [24, 25]. However, according to other studies of hospitalized patients, medications are a more common cause of anaphylaxis and fatal anaphylaxis [18–20]. These results show that the common causative agents of anaphylaxis vary depending on the study setting and study population. In this study, the bee sting was the most common cause of anaphylaxis-associated OHCA. Because Hymenoptera venom hypersensitivity is closely related to mast cell disorder, which often presents as anaphylaxis with vascular symptoms, OHCA can occur frequently in anaphylaxis caused by a bee sting. In the iatrogenic agents group, previous studies reported that platinum compounds and radiocontrast agents were the most common sources of drug-induced anaphylaxis in hospitals [26]. However, in this study, only three kinds of drugs were identified (anesthesia, antibiotics, and NSAIDS). They are all commonly used medications in the community. These results suggest that the most commonly used drugs are the most dangerous allergens of anaphylaxis-associated OHCA in the community.

When we analyzed the temporal trends of anaphylaxis-associated OHCA, we found that the arrests occurred most frequently during the day. This finding is consistent with previous findings reporting that most cardiac arrests occur during the daytime [16]. This U.S.-based study speculated that this trend occurred because nighttime arrests were often detected and reported in the morning [16]. However, in this study, this trend was because the bee stings, which accounted for the largest proportion of anaphylaxis-associated OHCAs, occurred during the day, when bees and people were active. Regarding seasonal variations, unlike the previous studies reporting that cardiac arrest occurred most frequently during the cold season, anaphylaxis-associated OHCAs occurred most frequently during summer (15). Usually, cardiovascular disease, including cardiac arrest, occurs more often during winter due to the effects of cold temperatures and the stress of the winter holiday season [16, 27]. However, for anaphylaxis-associated OHCA, exposure to the triggering agent is the most significant factor. Therefore, the peak season is different from those of arrests due to other causes. In cases of with bee stings, during the summer season, bees are most active, and humans are engaged in many outdoor activities. Furthermore, in Korea, there are traditions for weeding a grave in the mountains ahead Thanksgiving in autumn. Because of these factors, anaphylaxis-associated OHCA occurs frequently during summer. Seasonal variability was not apparent in the cases involving iatrogenic agents.

This study revealed that most of the anaphylaxis-associated OHCAs occurred during the day and during the summer season. This prevalence is mainly caused by bee stings in the natural agents group. Although cardiac arrest is associated with high mortality, anaphylaxis-associated OHCA can be avoided and effectively treated with an epinephrine injection in a timely manner. Therefore, avoiding dangerous allergens and advanced preparation are critical. To prevent the occurrence of anaphylaxis-associated OHCA, the health administrative agency and EMS personnel should be aware of when the anaphylaxis-associated arrest occurred and the identity of the dangerous allergen. Moreover, the implementation of campaigns and educational programs is necessary to avoid known allergens during dangerous seasons.

### Limitations

This study has several limitations. First, we captured anaphylaxis-associated OHCA cases from a record review of the national OHCA registry database. However, because identifying the exact cause of cardiac arrest was difficult, the cause of arrest may have been misclassified, and an anaphylaxis-associated OHCA may have been missed. Notably, if the arrest is not witnessed, determining whether the arrest was caused by anaphylaxis is difficult. The number of anaphylaxis-associated OHCAs may have been underestimated. Second, the causative agent of anaphylaxis was confirmed through an OHCA database review based on the ambulance work sheet and medical records. However, these records can be inaccurate, and sometimes, the causative agents are not recorded in the database. In this case, we conducted a manual review of the EMS cardiac arrest registry and medical record and estimated the causative agents through the location and circumstance of the arrest. Of 224 cases, 12 had anaphylaxis that occurred in the clinic or hospital, but no specific substance was recorded. We classified these cases as anaphylaxis by an iatrogenic agent. Careful attention should be paid to interpreting the results of this study in terms of causative agents. Third, in Korea, EMTs cannot legally administer epinephrine. Although epinephrine is critical to the management of anaphylaxis-associated OHCA, data on prehospital epinephrine use were not collected.

## Conclusions

The incidence of anaphylaxis-associated OHCA is considerably low and has significant temporal variability, with its peak during the day and during summer. Anaphylaxis-associated OHCA by natural agents was more common than by iatrogenic agents, but there was no difference in terms of the survival-to-discharge rate between the two groups.
